# Assessment of the Bacterial Loads of Vacuum-Thermoformed Removable Retainers (VTRR) in Patients under Retention Therapy: A Randomized Clinical Trial

**DOI:** 10.3390/healthcare10071239

**Published:** 2022-07-03

**Authors:** Andrea Butera, Simone Gallo, Maurizio Pascadopoli, Beatrice Sfondrini, Mario Alovisi, Anand Marya, Giulia Stablum, Andrea Scribante

**Affiliations:** 1 Unit of Dental Hygiene, Section of Dentistry, Department of Clinical, Surgical, Diagnostic and Pediatric Sciences, University of Pavia, 27100 Pavia, Italy; andrea.butera@unipv.it (A.B.); giulia.stablum01@universitadipavia.it (G.S.); 2 Unit of Orthodontics and Pediatric Dentistry, Section of Dentistry, Department of Clinical, Surgical, Diagnostic and Pediatric Sciences, University of Pavia, 27100 Pavia, Italy; beatrice.sfondrini01@universitadipavia.it; 3 Department of Surgical Sciences, Dental School, University of Turin, 0121 Turin, Italy; mario.alovisi@unito.it; 4 Department of Orthodontics, Faculty of Dentistry, University of Puthisastra, Phnom Penh 12211, Cambodia; amarya@puthisastra.edu.kh; 5 Center for Transdisciplinary Research, Saveetha Institute of Medical and Technical Science, Saveetha Dental College, Saveetha University, Chennai 600077, India

**Keywords:** orthodontics, retainers, retention, essix, microbiology, bacteria, periodontal indexes, randomized clinical trial, vacuum-thermoformed removable retainer

## Abstract

Retention devices are essential after orthodontic treatment in order to avoid the risk of relapse. For this goal, vacuum-thermoformed removable retainers (VTRRs) are useful tools in clinical practice. The main limitation related to them is the accumulation of plaque. The aim of this study was to investigate the bacterial loads present on VTRRs (Essix ACE Plastic, Dentsply Sirona) in patients under retention therapy. Patients were randomly divided into three groups, depending on the product used for the cleansing of the VTRR: Geldis, Polident tablets, and simple water, respectively. Microbiological samples were taken from the retainers at the baseline, after 1 and after 2 months, with the collection of Bleeding on Probing (BoP), Plaque Index (PI), Basic Erosive Wear Examination (BEWE) and Schiff Air Sensitivity test (SAI). A total of 15 patients were recruited and for each product, 5 patients were allocated. No significant intragroup and intergroup differences were observed at any time point for PI, SAI, BoP, Red Complex, Total Pathogen and Total Saprophyte loads. A significant intragroup and intergroup difference was assessed at T1 and T2 for BEWE in the control group. According to the results of this study, the bacterial load on VTRR retainers is not influenced by the cleaning methods tested.

## 1. Introduction

Several retention devices are used after orthodontic treatment in order to maintain arch form and minimize relapse [1]. Among the different possibilities, vacuum-formed retainers and Begg retainers are included; the former are preferred for aesthetics and comfort, the latter for functional capabilities such as chewing and biting [2]. A widely used device is also the lingual splint, mainly in the lower arch. This retainer, that is bonded to the lingual surfaces of the teeth, is increasingly preferred by orthodontists for being fixed and easy to tolerate by patients for long-term stay; however, there is a greater chance of accumulating plaque and tartar in the surrounding area [3] Among various types of removable retainers, the vacuum-thermoformed removable retainers (VTRR) were introduced in 1993 as an esthetic, comfortable and inexpensive alternative to traditional retainers. The considerable demand for preserving aesthetics during and after orthodontic treatment led to the development of devices that are almost invisible within the oral cavity. The limit of this retainer is that compliance is an essential requirement [4]. These types of removable appliances have other advantages such as comfort, reduction in chairside time, possibility of removing them both during meals and for the brushing of teeth such as clear aligners [5]. Despite these pros, costs are higher for vacuum-formed retainers [6,7].

However, orthodontic appliances in the oral cavity led to the possibility of having multiple sites of bacterial adhesion and formation of biofilms (plaque accumulation) with possible progression towards inflammatory processes such as gingivitis and periodontitis [8,9]. This biofilm coats both hard and soft tissues, it is composed of host constituents, bacteria, cell-free enzymes and polysaccharides [10]. The biofilm can mature with the adhesion and proliferation of periodontal pathogenic species including *S. mutans* [11]. If patients fail to maintain a good level of oral hygiene, the microorganisms incorporated in the plaque begin to proliferate and create a suitable habitat for hosting periodontal pathogenic species. [12]. For this reason, periodontal parameters such as plaque index (PI), gingival index (GI), bleeding on probing (BOP) and probing pocket depth (PPD) are evaluated [13]. In addition to the patient’s oral hygiene, plaque accumulation is also favored by the type of appliance. In subjects undergoing orthodontic therapy, the above indexes generally increase [13]. However, even thermoplastic appliances could facilitate bacterial adhesion if they have scratches and microabrations [14]. It was demonstrated that increased gingival inflammation and plaque scores were associated both with fixed mandibular retainer and vacuum-formed retainer in a 4-year follow up study [15].

Therefore, the aim of this study was to investigate the bacterial loads present on VTRR retainers in patients under retention phase using three different cleansing products. The first null hypothesis is that there are no significant intragroup and intergroup differences in the periodontal clinical indexes considering the three groups. Additionally, the second null hypothesis is that no significant differences occur for microbiological parameters.

## 2. Materials and Methods

### 2.1. Study Design and Participants

This is a randomized clinical trial with parallel assignment registered on ClinicaTrials.gov (NCT04871763). Patients referring to the Unit of Orthodontics and Pediatric Dentistry, Section of Dentistry, Department of Clinical, Surgical, Diagnostic and Pediatric Sciences, University of Pavia, Pavia, Italy for retention follow-up after the end of orthodontic therapy were recruited. Patients or their parents signed the informed consent before starting the trial. 

The inclusion criteria were: patients who completed orthodontic treatment and under VTRR phase. Exclusion criteria were: patients under orthodontic treatment with fixed appliances or clear aligners, non-orthodontic patients, and patients with a greater tendency to accumulate plaque (e.g., having congenital anomalies such as cleft lip and palate). 

### 2.2. Interventions and Outcomes

At the baseline (T0), participants underwent a professional supragingival and subgingival oral hygiene treatment using a piezoelectric and Gracey curettes. Patients were then instructed to correct domiciliary oral hygiene procedures, brushing their teeth for 2 min twice a day. VTRR (Essix ACE Plastic, Dentsply Sirona, Konstanz, Germany) had to be worn during sleep for all the nights. 

They were divided into 3 groups according to the type of cleaning of VTRR: in the first group, patients had to clean their VTRRs with Geldis^®^ Daily Cleanser gel with its specific toothbrush (Kalipharm srl, Milan, Italy); in the second group, VTRRs had to be left in a glass of water with one Polident tablet (GlaxoSmithKline, Brentford, England); in the third group, VTRRs had to be cleaned with a toothbrush under running water.

The composition of the products tested is shown in Table 1. 

Geldis was used applying a small amount of product on the VTRR. Then, a specific silicone-bristled toothbrush had to be used (in order to avoid scrapes), followed by rinsing with water. 

A Polident tablet was dropped into a glass with warm water, so that the VTRR could be completely covered. After soaking for 3–5 min, a soft brush had to be used. A rinsing of 1 min had to be performed after the use of the brush. 

The following periodontal indexes were evaluated: Plaque Control Record [16], Schiff Air Index [17], Bleeding on Probing [18] and BEWE index [19]. Microbiological evaluation was performed by means of a Real Time PCR-based test (BPA Basic, Biomolecular Diagnostic Srl, Firenze, Italy). The specific kit used allowed for the detection of the following bacteria: *Aggregatibacter actinomycetemcomitans*, *Porphyromonas gingivalis*, *Prevotella intermedia*, *Fusobacterium nucleatum*, Red Complex load, total pathogens load and total saprophytes load. Microbiological samples were collected with sterile papers, inserted for 30 s on VTRRs and then stored in a sterile test tube to be sent to the laboratory (storage at −20 °C). Patients were not asked to avoid food or beverage intake before sampling procedures.

Periodontal evaluation and microbiological samples were conducted at the baseline (T0), after 1 month (T1) and after 2 months (T2). 

### 2.3. Sample Size 

A sample size calculation was performed (alpha = 0.05; power = 80%) for two independent study groups and a continuous primary endpoint. Considering the variable Bleeding on Probing as the primary outcome, an expected mean of 15 was hypothesized, with a standard deviation of 7 [20]. The expected difference between the means was supposed to be 12; therefore, 5 patients were requested for each group. 

### 2.4. Randomization and Blinding 

Patients were randomly assigned to one of the three groups using a permuted block randomization table. An operator enrolled the participants and executed the professional oral procedures. Using previously prepared sequentially numbered, opaque, sealed envelopes (SNOSE), an assistant assigned patients to the respective treatment. The products were blinded so that the patients, the operator and the data analyst could be blinded too.

### 2.5. Statistical Methods 

Data were submitted to statistical analysis with R Software (R version 3.1.3, R Development Core Team, R Foundation for Statistical Computing, Wien, Austria). For each group and variable, the following descriptive statistics were calculated: mean, standard deviation, minimum, median, and maximum. PI was calculated in percentage; saprophytic and bacterial loads were calculated as percentages of the total microbiological loads; red complex was calculated as copies/microliter; SAI, BEWE index and BoP were calculated with the relative score. 

Data normality was assessed with Kolmogorov–Smirnov test. For each variable, inferential comparisons among groups were performed using ANOVA with post hoc Tukey test. Significance was predetermined for *p* < 0.05 for all of the statistical tests.

## 3. Results

**Figure 1:**
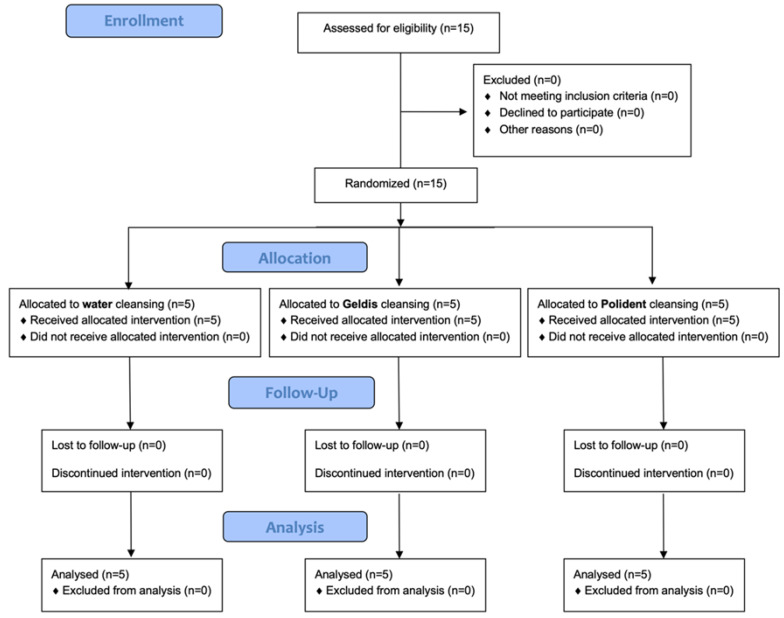
CONSORT flow chart of the study.

A total of 15 patients responding to the inclusion criteria were asked to participate in the study. They all agreed to participate and received the allocated interventions. No patient was excluded from the analysis. The flow chart of the study is shown in Figure 1. At baseline, the sample showed a mean age of 29.1 ± 9.1 years (11 females, mean age 29.3 ± 9; 4 males, mean age 28.5 ± 11).

Descriptive and inferential statistics are reported in the following sections. Inter- and intra-group comparisons are shown with letter-based comparisons [21].

### 3.1. Clinical Indexes

With regards to PI, SAI and BoP, no significant intergroup and intragroup differences were detected (*p* > 0.05) (Table 2, Table 3 and Table 4).

### 3.2. Microbiological Parameters

With regards to Total Pathogen, Total Saprophyte, and Red Complex, no significant intergroup and intragroup differences were detected (*p* > 0.05) (Table 5, Table 6 and Table 7).

## 4. Discussion

To avoid relapse, retainers are necessary at the end of orthodontic treatment in order to ensure the results remain as stable as possible. Retainers can be fixed or removable. The fixed ones, known as splints, are widely used but require a precise adhesion technique and a greater attention in performing correct oral hygiene procedures. The greater accumulation of plaque and tartar is related to a greater risk of developing caries and periodontal problems [3]. A study conducted by Levin et al. highlighted that splints are associated with an increase in the plaque index, gingival recession and bleeding on probing [22]. For these reasons, clinicians often choose a removable retainer. However, this retainer can also be associated with greater plaque accumulation [23]). On the basis of this evidence, it is necessary to define cleaning and disinfection protocols for thermoplastic appliances. 

The aim of this study was to investigate the bacterial loads present on VTRRs in patients under retention phase using three different cleansing products. 

The first null hypothesis was that there are no significant intragroup and intergroup differences in the periodontal clinical indexes considering the three groups. 

Additionally, the second null hypothesis was that no differences occur for microbiological parameters either. Both null hypotheses were rejected. No significant differences were generally assessed for neither periodontal nor clinical indexes, despite a better outcome for Geldis. In fact, this product contains an increased number of substances which might be responsible for this cleaning effect, such as sodium lauryl sulphate, cocamide dea, sodium chloride, mentha piperita oil, phenoxyethanol, benzoic acid, ascorbic acid, dehydroacetic acid, ethylhexyglycerin, and limonene. Even if insignificant, a higher reduction in bleeding indexes was assessed in the Geldis group.

Levrini et al.’s study showed that mechanical brushing of aligners associated with effervescent tablets leads to better results than just rinsing under water. Tablets allow a better cleaning result, but only if associated with mechanical brushing for the removal of the bacterial biofilm [23]. As demonstrated by Meto et al., the microbial biofilm is formed naturally on the surface of thermoplastic devices and despite washing with water and toothbrush the 3D images show the presence of microbial colonies. The association with Cupral (a compound based on copper-calcium hydroxide) exerts powerful effects against microbial plaque, naturally present on clear aligners. Its use has been innovative in orthodontics, while in previous clinical applications it was used in periodontology and endodontics [24]. Shpack et al. noted that there are regions of the thermoplastic appliance that have significantly greater biofilm adherence than the anterior region. In the oral cavity, plaque accumulates mainly on the vestibular surfaces of teeth, while the palatal ones have a self-cleansing effect thanks to the tongue. On the thermoformed device, it is just the opposite [14]. Other authors have shown that covering aligner surface with a coating of gold nanoparticles controlled the adhesion of the bacterial biofilm, in particular with the inhibition of *P. gingivalis* and *S. mutans* [25,26]. It is also important to take care of the appliance as both physical and chemical changes can lead to greater retention of plaque, so it is essential to instruct the patient to a correct cleansing of the VTRR [27]. 

The slight reduction in Geldis group microbiological parameters (except for the total saprophyte bacterial load) could be explained by recently investigated antimicrobial activity of mentha piperita essential oil [28].

According to Sfondrini et al., the use of these devices did not significantly affect the periodontal and microbiological parameters with respect to patients not under treatment [29]; similarly in our study time point, PI and BOP had no significant intragroup and intergroup differences, despite the fact that BOP was much superior at T2 in the control group which suggests the role of the experimental products in maintaining the periodontal status. The PCR index for the Water group increases in a non-statically significant way, for Polydent and Geldis at T2 the values decrease. BOP increases for the Water group between T0, T1 and T2; for Geldis it decreases; while for Polident between T0 and T1 it increases and between T1 and T2 it decreases. As regards SAI score, a reduction was assessed for all the groups at T2 which is probably related to the decrease in sensitivity for all of the patients who get used to wear the VTRRs. 

Instead, for both the control and the trial group, the total bacteria count was significantly increased compared to values, respectively, assessed at T0 and at T1 (intragroup differences), no significant intergroup differences were assessed. In the same way, the results here obtained show that Red Complex values are reduced or remain the same as the initial values. Despite this being insignificant, a reduction for the Red Complex load was generally assessed in the groups at T1 with a certain increase at T2. It could be hypothesized that the cleaning methods of the VTRR exert a major effect on these bacteria especially at a short term. 

Schiff Air Index remains almost constant between T0, T1 and T2. BEWE index showed significant differences for the Water group whose values increase, while the values of Geldis and Polydent remain almost constant in the 3 timeframes. 

In order to carry out the microbiological evaluation, a commercial kit was used as it has been in previous recent research [18]. However, due to the fact that *Tannerella forsythia* and *Treponema denticola* are excluded from the microbiological assessment of the BPA Basic kit used in this study, further analysis should also include these bacterial species. Moreover, for a more precise and complete analysis, the kit should identify which species were included in “saprophytic bacterial loads”.

The limitations of this report are the short follow-ups (1 and 2 months) and that only patients belonging to the department could participate in the study. Moreover, only one category of VTRR was tested, but further types of these devices should be considered to detect any different effects on periodontal health between them (it could compare the effects of other types of removable retainer such as Positioners, Spring retainers or other resin appliance). The fact that no specific toothbrush was given to patients for domiciliary oral hygiene is another limitation of the study. Moreover, it would be interesting to evaluate how different electric toothbrushes could influence the clinical and microbiological parameters taken into account. Additionally, the products tested here should be compared with other substances available on the market. Finally, it could also be interesting to assess whether the same effects of this study would be obtained considering a different timing of cleaning. 

## 5. Conclusions

The correct cleaning of VTRRs is a fundamental aspect. On the basis of the results of this study, no significant differences for microbiological and periodontal clinical parameters were generally assessed considering the three cleaning methods here tested. 

## Figures and Tables

**Table 1:** Composition of the tested products.

Product	Manufacturer	Composition
Geldis Daily Cleanser	Kalipharma Srl, Como (CO), Italy	aqua, sodium laureth sulphate, cocamide DEA, sodium chloride, mentha piperita oil, phenoxyethanol, benzoic acid, ascorbic acid, dehydroacetic acid, ethylhexyglycerin, limonene
Polident Intense Freshness	GlaxoSmithKline S.p.A., Verona (VR), Italy	sodium bicarbonate, citric acid, potassium caroate (potassium monopersulphate), sodium carbonate, sodium carbonate peroxide, TAED, sodium benzoate, PEG-180, sodium lauryl sulphate, VP/VA copolymer, aroma, subtilisin, cellulose gum, CI 42090, CI 73015

**Table 2:** Descriptive statistics of Plaque Control Record measurements (PCR%).

Group	Time	Mean	St Dev	Min	Median	Max	Significance *
Water	T0	38.00	32.72	0.00	29.00	79.00	A
	T1	51.20	27.62	22.00	59.00	78.00	A
	T2	72.00	18.84	41.00	73.00	89.00	A
Geldis	T0	40.80	21.51	16.00	46.00	65.00	A
	T1	42.60	16.50	17.00	43.00	62.00	A
	T2	36.60	22.03	7.00	42.00	65.00	A
Polident	T0	53.80	16.18	38.00	45.00	75.00	A
	T1	65.60	18.58	47.00	66.00	90.00	A
	T2	64.40	26.03	30.00	67.00	91.00	A

* Different letters show statistically significant differences among the groups (*p* < 0.05).

**Table 3:** Descriptive statistics of Schiff Air Index measurements.

Group	Time	Mean	St Dev	Min	Median	Max	Significance *
Water	T0	0.60	0.89	0.00	0.00	2.00	A
	T1	0.60	0.89	0.00	0.00	2.00	A
	T2	0.40	0.89	0.00	0.00	2.00	A
Geldis	T0	1.00	0.71	0.00	1.00	2.00	A
	T1	1.00	0.71	0.00	1.00	2.00	A
	T2	0.60	0.55	0.00	1.00	1.00	A
Polident	T0	1.00	0.71	0.00	1.00	2.00	A
	T1	1.00	1.22	0.00	1.00	3.00	A
	T2	0.80	1.10	0.00	0.00	2.00	A

* Different letters show statistically significant differences among the groups (*p* < 0.05).

**Table 4:** Descriptive statistics of Bleeding on Probing measurements.

Group	Time	Mean	St Dev	Min	Median	Max	Significance *
Water	T0	1.40	2.61	0.00	0.00	6.00	A,B
	T1	1.60	1.14	0.00	2.00	3.00	A
	T2	3.20	3.11	0.00	4.00	7.00	A,B
Geldis	T0	1.20	2.17	0.00	0.00	5.00	A,B
	T1	0.20	0.45	0.00	0.00	1.00	A,B
	T2	0.20	0.45	0.00	0.00	1.00	A,B
Polident	T0	1.20	1.79	0.00	0.00	4.00	A,B
	T1	2.40	3.36	0.00	1.00	8.00	A,B
	T2	0.00	0.00	0.00	0.00	0.00	B

* Different letters show statistically significant differences among.

**Table 5:** Descriptive statistics of Total Pathogen loads measurements.

Group	Time	Mean	St Dev	Min	Median	Max	Significance *
Water	T0	5.80	8.01	0.00	0.00	16.00	A
	T1	2.70	5.98	0.00	0.00	13.40	A
	T2	2.40	5.37	0.00	0.00	12.00	A
Geldis	T0	0.03	0.08	0.00	0.00	0.17	A
	T1	0.30	0.67	0.00	0.00	1.50	A
	T2	3.61	4.82	0.02	2.21	12.00	A
Polident	T0	4.40	7.16	0.00	2.00	17.00	A
	T1	0.00	0.00	0.00	1000	0.00	A
	T2	4.44	6.30	0.00	1.80	15.00	A

* Different letters show statistically significant differences among the groups (*p* < 0.05).

**Table 6:** Descriptive statistics of Total Saprophyte loads measurements.

Group	Time	Mean	St Dev	Min	Median	Max	Significance *
Water	T0	94.20	8.01	84.00	100.00	100.00	A
	T1	97.38	5.80	87.00	100.00	100.00	A
	T2	97.52	5.55	87.60	100.00	100.00	A
Geldis	T0	99.96	0.09	99.80	100.00	100.00	A
	T1	99.70	0.67	98.50	100.00	100.00	A
	T2	96.26	5.01	87.50	97.80	99.90	A
Polident	T0	95.60	7.16	83.00	98.00	100.00	A
	T1	100.00	0.00	100.00	100.00	100.00	A
	T2	95.60	6.27	85.00	98.00	100.00	A

* Different letters show statistically significant differences among.

**Table 7:** Descriptive statistics of Red Complex loads measurements.

Group	Time	Mean	St Dev	Min	Median	Max	Significance *
Water	T0	0.20	0.45	0.00	0.00	1.00	A
	T1	0.00	0.00	0.00	0.00	0.00	A
	T2	0.20	0.45	0.00	0.00	1.00	A
Geldis	T0	0.00	0.00	0.00	0.00	0.00	A
	T1	0.00	0.00	0.00	0.00	0.00	A
	T2	0.00	0.00	0.00	0.00	0.00	A
Polident	T0	0.40	0.55	0.00	0.00	1.00	A
	T1	0.00	0.00	0.00	0.00	0.00	A
	T2	0.20	0.45	0.00	0.00	1.00	A

* Different letters show statistically significant differences among.

## Data Availability

Data are available upon reasonable request from the corresponding author.
